# Cycloserine-induced neuropsychiatric toxicity in multidrug-resistant tuberculosis: association with peak plasma concentration

**DOI:** 10.1128/spectrum.01437-25

**Published:** 2025-11-05

**Authors:** Junjie Cheng, Jinmeng Li, Ren Zheng, Kan Xu, Xiaoqing Ma, Junke Qiu, Ruoying Zhang, Xinjun Cai

**Affiliations:** 1 Department of Pharmacy, Zhejiang Hospital of Integrated Traditional Chinese and Western Medicine, Hangzhou, China; 2 Department of Tuberculosis, Zhejiang Hospital of Integrated Traditional Chinese and Western Medicine, Hangzhou, China; University of Kentucky, Lexington, Kentucky, USA

**Keywords:** cycloserine, peak plasma concentration, neuropsychiatric-related adverse drug reactions

## Abstract

Cycloserine (Cs) is a crucial anti-multidrug-resistant tuberculosis medication with significant clinical efficacy. However, its clinical application is limited by neuropsychiatric-related adverse drug reactions (ADRs). This study retrospectively evaluated the peak plasma concentration (*C*_max_) of Cs and Cs-related neuropsychiatric ADRs and analyzed the risk factors of developing neuropsychiatric ADRs. A multivariable ordinal logistic regression model was built to predict the incidence of neuropsychiatric ADRs. This study enrolled a total of 136 participants, with a cumulative incidence of neuropsychiatric ADRs of 12.5%. Patients experiencing ADRs had significantly higher Cs *C*_max_ compared to those without ADRs. The risk of ADRs rose with increasing Cs exposure, especially when *C*_max_ exceeded 30 mg/L. Receiver operating characteristic analysis demonstrated the strong predictive value of *C*_max_ for neuropsychiatric ADRs. Notably, the predictive efficacy of the logistic regression model significantly improved after incorporating drinking history as a covariate, achieving a final predictive accuracy of 87.6%. Cs *C*_max_ serves as a critical indicator for predicting Cs-related neuropsychiatric ADRs. Dynamic monitoring of Cs *C*_max_ combined with alcohol history assessment can enable accurate identification of high-risk populations for neuropsychiatric ADRs, thereby providing a scientific basis for developing individualized medication regimens and implementation of early interventions.

**IMPORTANCE:**

Cycloserine (Cs) is a vital medication for treating multidrug-resistant tuberculosis (MDR-TB), but its use is often limited by severe neuropsychiatric side effects such as depression, anxiety, and seizures. This study highlights the critical role of monitoring Cs peak plasma concentration (*C*_max_) to predict and mitigate these adverse reactions. By analyzing data from 136 patients, the researchers found that higher Cs *C*_max_ levels significantly increase the risk of neuropsychiatric toxicity, particularly when concentrations exceed 30 mg/L. Additionally, a history of alcohol consumption further elevates this risk. These findings provide a practical approach for clinicians: regular monitoring of Cs levels and assessing alcohol use can help identify high-risk patients early, enabling timely adjustments to treatment plans. This work not only enhances patient safety but also supports the broader goal of improving MDR-TB treatment outcomes by minimizing harmful side effects.

## INTRODUCTION

Multidrug-resistant tuberculosis (MDR-TB) is defined as *Mycobacterium tuberculosis* infection that is resistant to isoniazid and rifampicin, the two most powerful anti-TB drugs (1). Emergence of drug-resistant strains is the leading cause of MDR-TB and chemotherapy failure (2). Globally, only 60% of the MDR-TB cases have been successfully treated with 15% of patients still dying from the disease (3). Cycloserine (Cs) is a second-line anti-TB drug that inhibits the growth of *M. tuberculosis* through the competitive inhibition of d-alanine-abrogating enzymes and synthetases during bacterial peptidoglycan synthesis (4, 5). In the treatment of MDR-TB, Cs was found to be highly effective, with minimal drug resistance and no cross-resistance to other anti-TB drugs (6, 7), and it is often used in combination with linezolid and moxifloxacin (8). The addition of Cs to treatment regimens increases the effectiveness of MDR-TB treatment and reduces mortality (9). Therefore, the World Health Organization classified Cs as a group B drug for the treatment of MDR-TB and recommends its use in long-term treatment regimens (3).

However, Cs is associated with an increased susceptibility to severe neuropsychiatric adverse drug reactions (ADRs) during the treatment of MDR-TB, with the most prominent adverse effects being headache, depression, anxiety, and other psychiatric disorders. These side effects greatly hinder the clinical utility of Cs and can require discontinuation of treatment in approximately 9% of patients (10, 11). Given the considerable burden and increased number of cases of MDR-TB, understanding the pharmacokinetics and safety of Cs in patients with MDR-TB is of critical importance.

This study evaluated the degree of Cs-induced neuropsychiatric ADRs and tracked the plasma concentration at 2 h post-administration of the Cs (defined as *C*_max_) in patients with MDR-TB. Our aim was to analyze the relationship between Cs *C*_max_ and the incidence of neuropsychiatric ADRs and to develop a predictive model for identifying high-risk populations for neuropsychiatric ADRs.

## MATERIALS AND METHODS

### Study design and patient recruitment

This retrospective cohort study enrolled patients diagnosed with MDR-TB who received treatment at the Zhejiang Hospital of Integrated Traditional Chinese and Western Medicine between January 2020 and December 2022. This study was conducted in accordance with the Declaration of Helsinki (revised 2013) and approved by the Medical Ethics Committee of Zhejiang Hospital of Integrated Traditional Chinese and Western Medicine (No. [2023] trials (053)). Written informed consent was obtained from each patient in this study. Patient data were collected anonymously to ensure confidentiality of their information. Throughout the therapeutic course, Cs treatment regimen was dynamically formulated and meticulously adjusted based on ongoing clinical assessments, including radiographic findings, microbiological results, and drug susceptibility testing. These decisions were made by a multidisciplinary TB specialist team, ensuring an individualized and optimized therapeutic approach.

The inclusion criteria were as follows: age >18 years, diagnosis of MDR-TB, inclusion of Cs in the treatment regimen, and regular use of Cs to achieve a steady-state plasma concentration (at least three consecutive days). The exclusion criteria included failure on the Cycloserine Neuropsychiatric Adverse Reaction Assessment Scale at admission, a history of epilepsy, depression, or anxiety.

To evaluate potential selection bias, we compared baseline characteristics between included and excluded patients. Among the 206 initially screened MDR-TB patients, 70 were excluded: 52 due to incomplete pharmacokinetic or follow-up data, 12 with pre-existing neuropsychiatric disorders, and 6 with irregular Cs administration. Comparative analysis showed no significant differences in age, sex, or clinical disease severity between included and excluded groups, suggesting that exclusions did not substantially introduce bias into the final cohort.

### Data collection and therapeutic drug monitoring (TDM)

Demographic characteristics and clinical data were collected from case histories, including age, sex, body mass index (BMI), smoking history, alcoholism, comorbidities (hepatitis B, diabetes mellitus, and hypertension), diagnosis (pulmonary TB, extrapulmonary TB, and pulmonary TB with extrapulmonary TB), background regimen (linezolid, clofazimine, etc.), daily dose of Cs, and *C*_max_.

TDM was usually performed at 7, 14 days, and monthly follow-up visits after the first dose of Cs. Neuropsychiatric evaluations were implemented when clinically indicated, based on observed behavioral changes or reported symptoms. However, a complete monitoring schedule could not be maintained for all the participants due to difficulties in sustaining consistent follow-up and variations in patient situations. Blood samples were collected at 2 h after dosing (details below) and analyzed using high-performance liquid chromatography-mass spectrometry following the standard procedure as previously reported (12). According to the NHS Labor Antimicrobial Reference Laboratory, the recommended range of Cs *C*_max_ in patients with MDR-TB is 20–35 mg/L (13).

### Definition of steady-state Cs *C*_max_

For patients undergoing conventional Cs (Zhejiang Hisun Pharmaceutical Co., Ltd.) therapy (defined as oral administration of 250 mg twice or thrice daily for a minimum of three consecutive days), pharmacokinetic steady-state conditions were considered. In these cases, TDM was performed precisely 2 h after the subsequent scheduled dose administration, with the measured concentration representing the *C*_max_. This sampling protocol was implemented based on established pharmacokinetic principles, indicating that a minimum of 3 days of consistent dosing is required to achieve steady-state concentrations of Cs and that the 2-h post-dose interval corresponds to the characteristic absorption peak of the drug in clinical studies (14).

### Definition of Cs-induced neuropsychiatric toxicity

Prior to initiating Cs therapy, each patient was advised of the expected toxic effects and evaluated for psychoneurological status according to the “Cycloserine Neuropsychiatric Adverse Reaction Assessment Scale” (Table S1) (15). Only patients who met predefined psychoneurological safety criteria were deemed eligible for Cs treatment. The assessment scale and Common Terminology Criteria for Adverse Events (CTCAE, v5.0) were administered when patients had behavioral changes or reported symptoms. ADRs were classified and graded according to the standardized CTCAE as grade 1, 2, or 3 corresponding to mild, moderate, or severe, respectively (no grade 4 or 5 was observed in this study). Any confirmed case of neuropsychiatric toxicity necessitated immediate dose adjustment or permanent discontinuation of Cs therapy as the expert team recommended.

### Statistical analyses

All statistical analyses were performed using SPSS (version 22.0; IBM Corp., Armonk, NY, USA) and Graphpad Prism 10.0 (GraphPad Software, San Diego, CA, USA). Continuous variables with a normal distribution are expressed as mean ± standard deviation (mean ± SD), while non-normally distributed variables are expressed using median with interquartile ranges (IQRs). Comparisons between two groups were performed using Student’s *t*-test (normally distributed) and non-parametric Mann–Whitney *U* rank-sum test (non-normally distributed). Categorical variables are expressed as percentages, and comparisons among groups were performed using the chi-square test or Fisher’s exact probability test. Statistical significance was set at *P* < 0.05.

## RESULTS

### Patient characteristics

In total, 136 patients with MDR-TB and 322 concentration measurements were included in this study. The baseline demographic and clinical characteristics are shown in Table 1. The median age of the included patients was 39 years (IQR: 27–58 years) with 86 males and 50 females. Cs was always combined with linezolid and clofazimine (81.62%) to treat MDR-TB. The majority of patients (61.67%) received Cs treatment at 500 mg per day and the median *C*_max_ of Cs was 19.3 mg/L (IQR: 14.49–24.98 mg/L).

**TABLE 1:** Characteristics of MDR-TB patients^*a*^

Characteristics	Total (*n* = 136)
Age, years	39 (27, 58)
Sex, male/female	86/50
BMI	19.40 (17.75, 21.95)
Smoking	44 (32.35%)
Alcohol consuming	22 (16.18%)
Comorbidities	
Hepatitis B	10 (7.35%)
Diabetes mellitus	18 (13.24%)
Hypertension	16 (11.76%)
Diagnosis	
Pulmonary tuberculosis	103 (75.74%)
Extrapulmonary tuberculosis	5 (3.68%)
Pulmonary and extrapulmonary tuberculosis	28 (20.59%)
Background regimen	
Linezolid	111 (81.62%)
Clofazimine	111 (81.62%)
Bedaquiline	57 (41.91%)
Pyrazinamide	53 (38.97%)
Levofloxacin	50 (36.76%)
Prothionamide	43 (31.62%)
Moxifloxacin	33 (24.26%)
Amikacin	19 (13.97%)
Delamanid	14 (10.29%)
Ethambutol	7 (5.15%)
P-aminosalicylic acid	7 (5.15%)
Capreomycin	1 (0.73%)
Cycloserine treatment and *C*_max_	
Initiated dose 500 mg/day	84 (61.76%)
Initiated dose 750 mg/day	52 (38.24%)
*C*_max_ (mg/L)	19.30 (14.49, 24.98)
*C*_max_ range	
<20 mg/L	176 (54.15%)
20–30 mg/L	110 (33.85%)
>30 mg/L	36 (11.08%)

^*a*^ BMI, body mass index; *C*_max_, peak plasma concentration.

### Neuropsychiatric ADRs associated with CS treatment

**Fig 1:**
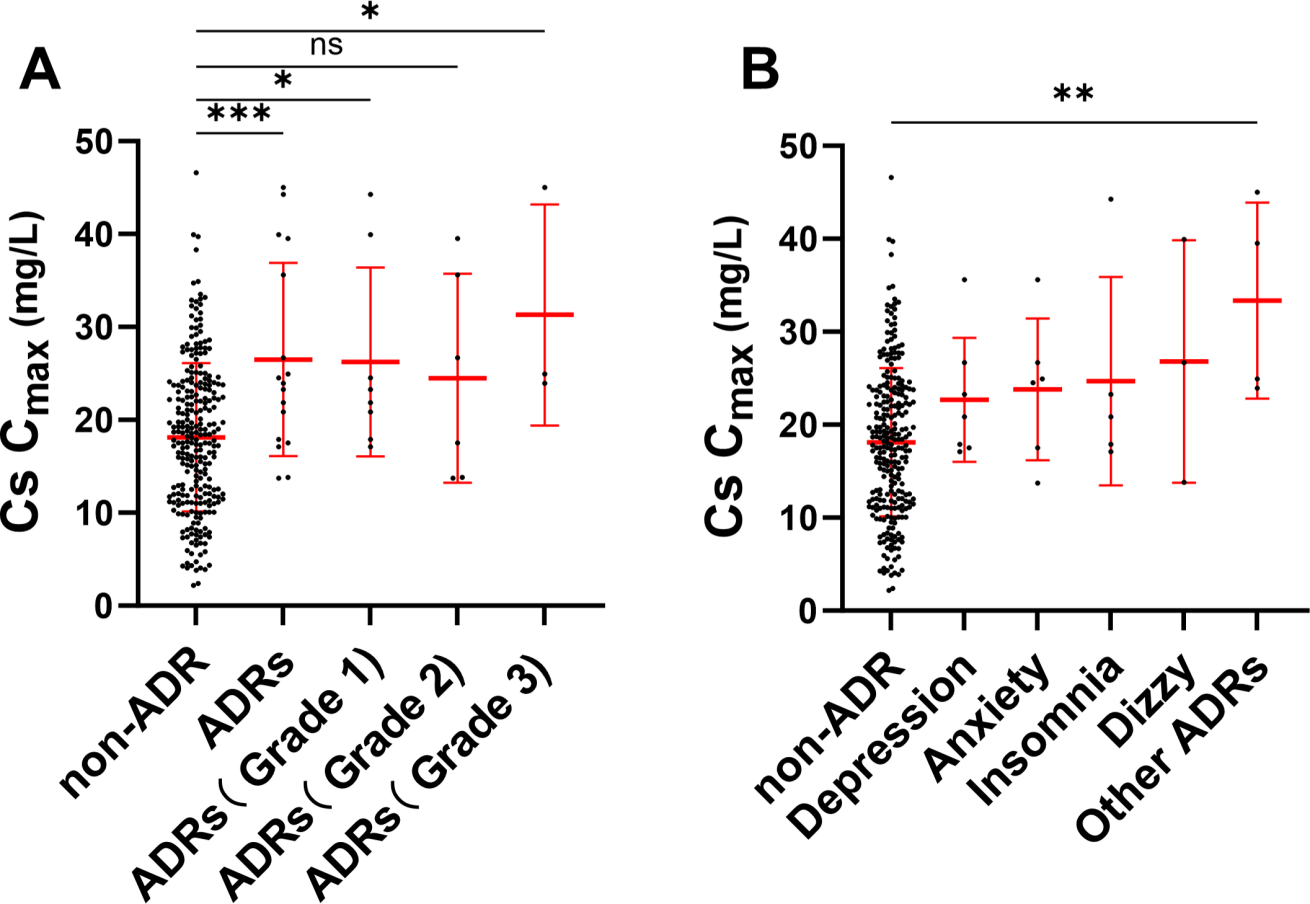
Association between Cs *C*_max_ and neuropsychiatric ADRs. (**A**) Comparison of Cs *C*_max_ (mg/L) between patients with neuropsychiatric ADRs (any grade) and those without ADRs. (**B**) Cs *C*_max_ (mg/L) values across different neuropsychiatric ADRs and non-ADRs groups. Data are presented as mean ± SD, *, *P*＜0.05. **P ＜ 0.01. ***, *P*＜0.001, ns indicates no significance (*P* > 0.05), compared to the non-ADR group. Note: other ADRs including seizure, tremor, delusion, and mania. ADRs, adverse drug reactions; *C*_max_, peak plasma concentration; Cs, cycloserine.

Of the 136 patients, 12.5% (17/136) experienced a neuropsychiatric ADR at least once. Based on the CTCAE, the severity distribution of neuropsychiatric ADRs was as follows: 47.06% (8/17) grade 1, or mild; 35.29% (6/17) grade 2, or moderate; and 17.65% (3/17) grade 3, or severe (Table 2). Patients who experienced neuropsychiatric ADRs had a significantly greater Cs *C*_max_ (26.50 mg/L vs 18.11 mg/L, *P* = 0.0003) compared to patients who did not experience ADRs (Fig. 1A). When stratified by CTCAE severity grades, patients with grade 3 ADRs demonstrated the highest Cs *C*_max_ levels (31.29 ± 4.80 mg/L), followed by grade 1 (26.22 ± 2.97 mg/L) and grade 2 (24.47 ± 3.42 mg/L) cases. The most common ADRs were depression (41.18%, 7/17) and anxiety (35.29%, 6/17). However, no statistically significant differences in Cs *C*_max_ were observed between the depression subgroup (22.70 ± 3.15 mg/L) and anxiety subgroup (23.82 ± 3.08 mg/L) compared to the non-ADR group (18.11 ± 3.87 mg/L). In contrast, patients experiencing seizure, tremor, delusion, and mania, which are defined as other ADRs in Fig. 1B, exhibited significantly elevated Cs *C*_max_ levels (33.35 ± 4.02 mg/L vs 18.11 ± 3.87 mg/L, *P* = 0.001) (Fig. 1B). These results indicated that the *C*_max_ of Cs is strongly correlated with neuropsychiatric ADRs.

**TABLE 2:** ADRs classification during Cs treatment

Variable	Patients (*n* = 17)
Depression	7 (41.18%)
Anxiety	6 (35.29%)
Insomnia	5 (29.41%)
Dizzy	3 (17.65%)
Seizure	1 (5.89%)
Tremor	1 (5.89%)
Delusion	1 (5.89%)
Mania	1 (5.89%)

### Incidence and timeline of Cs-induced neuropsychiatric ADRs

**Fig 2:**
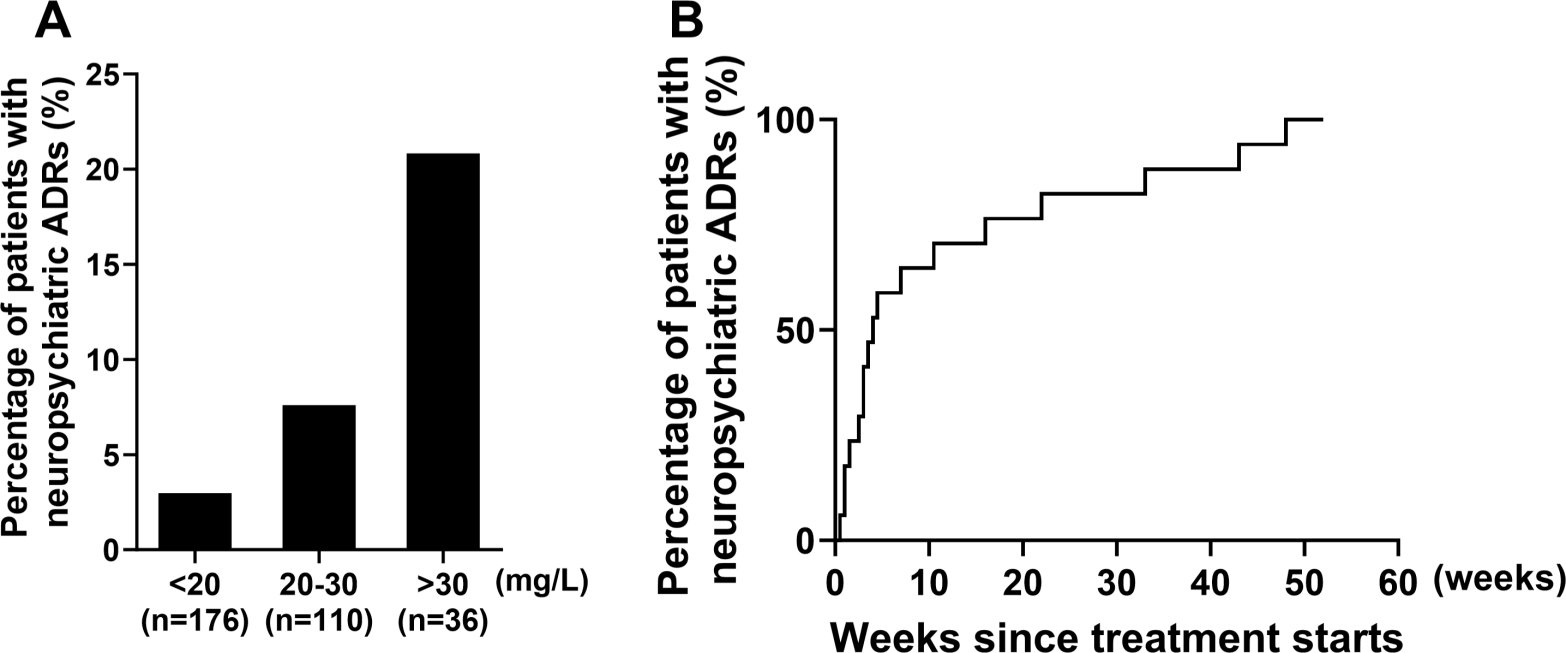
Incidence and timing of Cs-induced neuropsychiatric ADRs. (**A**) Incidence of Cs-induced neuropsychiatric ADRs. (**B**) Time-to-onset (days) of neuropsychiatric ADRs after initiation of Cs therapy. ADRs, adverse drug reactions; Cs, cycloserine.

Our findings demonstrated a concentration-dependent relationship, with the incidence of neuropsychiatric ADRs progressively increasing with higher Cs exposure levels. Specifically, patients with a *C*_max_ exceeding 30 mg/L exhibited a 20.88% incidence rate of neuropsychiatric toxicity, representing a significant increase compared to those maintaining a *C*_max_ below this threshold (Fig. 2A). The median time to onset of neuropsychiatric ADRs was 4 (3, 16) weeks (Fig. 2B). Notably, all documented ADRs resolved completely within 1–4 weeks following Cs discontinuation, with no recurrence or persistent sequelae observed during subsequent follow-up.

### Predictive thresholds for *C*_max_ in Cs-induced neuropsychiatric ADRs

**Fig 3:**
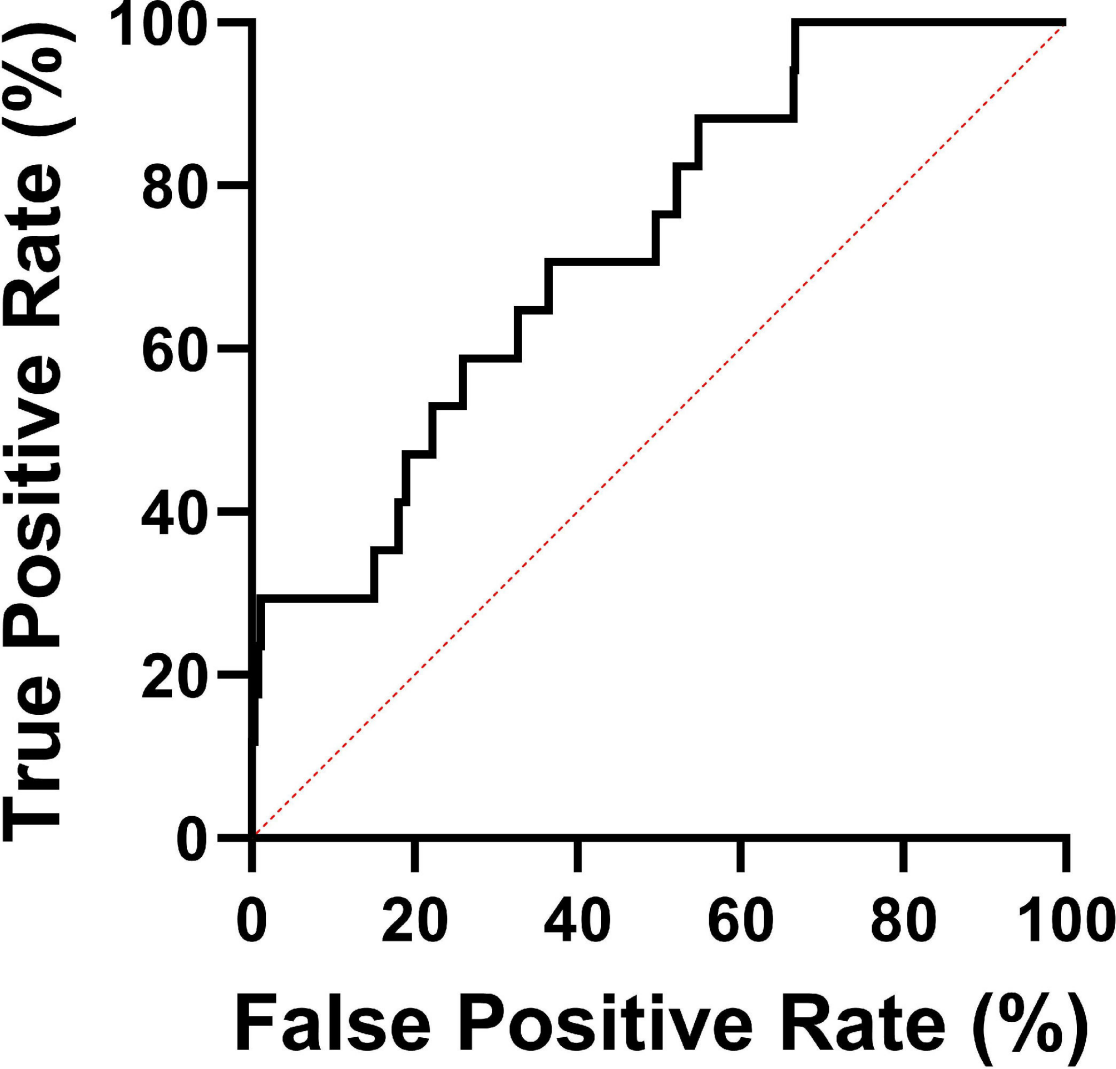
ROC curve for predicting neuropsychiatric toxicity of Cs *C*_max_. The AUC is 0.726, with an optimal *C*_max_ cut-off value of 23.27 mg/L, yielding a sensitivity of 70.59% and specificity of 63.53%. AUC, area under the curve; ROC, receiver operating characteristic.

To evaluate the predictive potential of Cs *C*_max_ for neuropsychiatric ADRs, we performed a receiver operating characteristic (ROC) curve analysis. The results identified a critical threshold of *C*_max_ > 23.24 mg/L (sensitivity: 70.59% and specificity: 63.53%) as a significant predictor for neuropsychiatric ADRs, demonstrating moderate predictive accuracy with an area under the curve (AUC) of 0.726 (95% confidence interval [CI]: 0.612–0.840; *P* = 0.0018) (Fig. 3).

### Logistic analysis of risk factors and predictive model for Cs-induced neuropsychiatric ADRs

**Fig 4:**
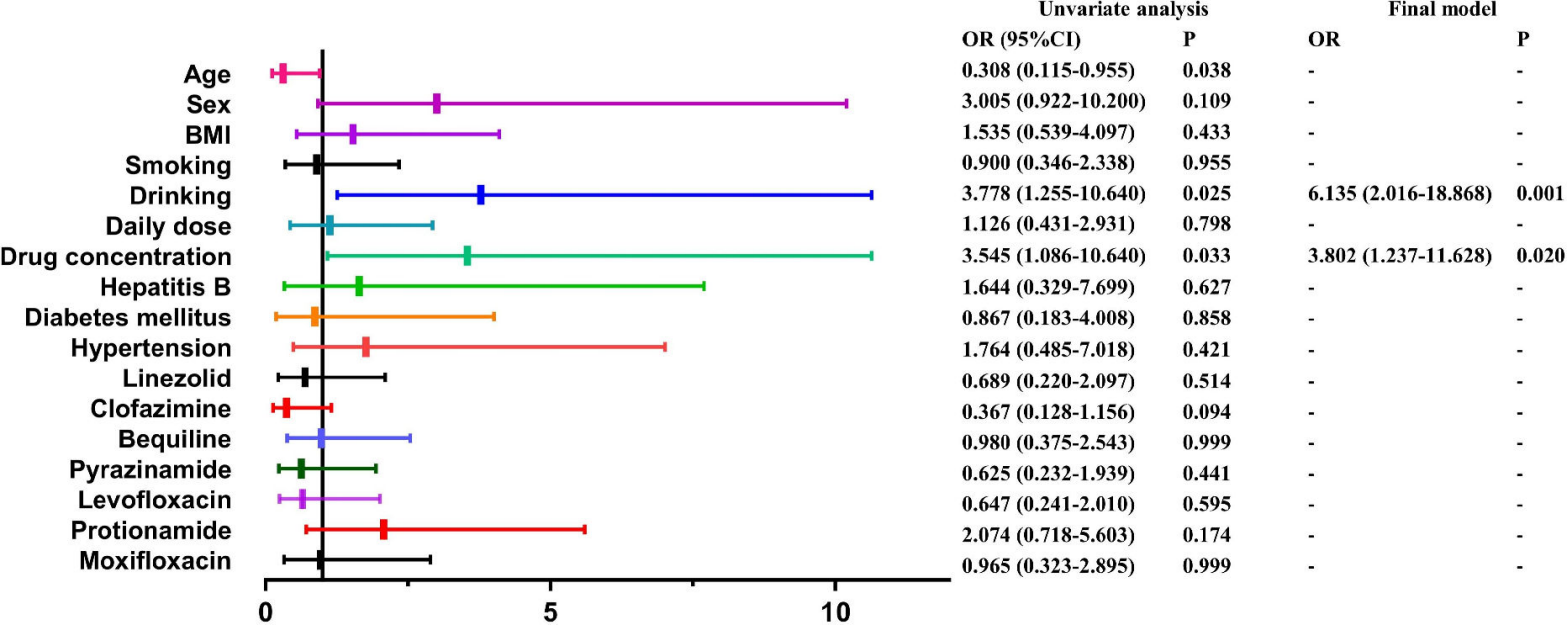
Logistic regression analysis of factors associated with Cs-induced neuropsychiatric ADRs. BMI, body mass index; *C*_max_, peak plasma concentration; Cs, cycloserine.

We performed a comprehensive multifactorial analysis and established a logistic regression model to develop a more precise predictive model for Cs-induced neuropsychiatric ADR (Fig. 4). Our findings demonstrate that Cs *C*_max_ is a significant risk factor for neuropsychiatric toxicity (*P* = 0.0325). Additionally, individuals over 60 years of age (*P* = 0.0379) and those with a history of alcohol consumption (*P* = 0.0249) were identified as being at an elevated risk for neuropsychiatric toxicity. Using reverse stepwise binary logistic regression modeling, alcohol consumption (odds ratio [OR] = 6.1350, *P* = 0.001) and *C*_max_ (OR = 3.8022, *P* = 0.020) were identified as independent risk factors for neuropsychiatric ADRs. The model exhibited excellent goodness-of-fit, as evidenced by the Hosmer and Lemeshow test (*χ*^2^ = 1.148, degrees of freedom [df] = 2, *P* = 0.563) and robust explanatory power, indicated by a Nagelkerke *R*^2^ value of 0.218. The overall predictive accuracy of the model is 87.6%.

## DISCUSSION

Cs has been used for more than 30 years to treat MDR-TB. Patients with MDR-TB treated with Cs-containing medications have a 66–77% success rate, with minimal rates of death and recurrence (17). However, Cs is associated with a greater frequency of neuropsychiatric ADRs than other anti-TB drugs, and the reported incidence of ADRs varies greatly between studies. A total of 623 patients with MDR-TB receiving Cs treatment were included in a study, which revealed that the occurrence of neuropsychiatric ADRs was 4.3% (18). Another cohort study observed that 9.28% (22/237) of MDR-TB patients experienced neuropsychiatric ADRs, and 45.25% of patients exhibited symptoms of pathological depression, such as low mood and cognitive slowing (19). Other studies have revealed more serious safety concerns during MDR-TB treatment, with 38% of the patients experiencing at least one Cs-induced neuropsychiatric ADR during treatment, characterized by neuropathy, depression, and psychosis (20). In the current study, 12.5% (17/136) of patients had at least one neuropsychiatric ADR, especially depression and anxiety. More than half of the patients experienced neuropsychiatric ADRs shortly after Cs treatment (within 4 weeks), which is partly consistent with the findings of previous studies. A cohort study reported that half of the psychiatric disorders in patients were detected within 8 weeks of Cs therapy (19). Another study reported that 44.4% of patients experienced psychiatric symptoms of varying severity within the first 8 weeks of Cs treatment (16).

Cs is rapidly and fully absorbed orally, with high bioavailability (70–90%) and widespread tissue distribution. The renal system is the primary pathway for Cs excretion. Studies have indicated that the combination of Cs with ethionamide, prothionamide, isoniazid, or fluoroquinolones may lead to heightened neuropsychiatric toxicity (21). Individuals with a history of alcohol consumption may have heightened susceptibility to convulsive seizures due to the suppressive effects of alcohol on the convulsion threshold (22, 23). Therefore, clinical guidelines recommend monitoring the blood concentration of Cs, particularly when co-administered with ethionamide, prothionamide, or fluoroquinolones (24). In this study, Cs *C*_max_ values were measured several times in each patient. Notably, a substantial proportion of patients did not reach the therapeutic window, which is consistent with a previous study indicating that 54.87% of patients received suboptimal concentrations of Cs therapy (22). In addition, we found that patients with neuropsychiatric ADRs had a much higher Cs *C*_max_ than those without ADRs, and these individuals tended to have a very high Cs *C*_max_ when they experienced severe ADRs.

ROC curve analysis demonstrated that Cs *C*_max_ > 23.27 mg/L could predict neuropsychiatric toxicity, with 70.59% sensitivity and 63.53% specificity. These results suggest that if Cs *C*_max_ was considered the only predictor of neuropsychiatric toxicity, it would correctly identify 70.59% of patients. However, the model overestimated the occurrence of neuropsychiatric ADRs in 36.47% of the population. This is not a perfect positive correlation with neuropsychiatric toxicity; therefore, Cs *C*_max_ measurements should be combined with other clinical factors to avoid the risk of neuropsychiatric toxicity.

Furthermore, logistic regression analysis indicated that neuropsychiatric ADRs were more common in patients older than 60 years, which is contrary to the findings of previous studies. A Chinese study that included 237 patients under Cs treatment showed that patients aged <40 years were at a higher risk of depression, which may be due to greater social pressure in young, middle-aged people (19). We believe that our results may be related to the direct measurement of Cs *C*_max_ because the elderly (>60 years) may have a poor renal clearance rate, which leads to drug accumulation. Subsequent model analysis also showed that age and *C*_max_ were collinear; thus, age was excluded from the final model.

A history of alcohol consumption was another factor affecting Cs-related neuropsychiatric ADRs in the final model. As early as 1965, studies showed that alcoholism is a risk factor for Cs-induced psychosis (25). Alcohol reduces the convulsive threshold, which may put alcoholics at risk of convulsive seizures (26). Additionally, clinical research has demonstrated a higher risk of seizures in individuals with alcoholic hepatitis (27). In our study, patients with a history of alcohol consumption were 6.135 times more likely to experience Cs-induced neuropsychiatric ADRs than non-drinkers. Hence, patients with MDR-TB and a history of alcohol consumption who receive Cs treatment should have their *C*_max_ constantly monitored to prevent Cs-induced neuropsychiatric ADRs.

### Limitations

This study has several limitations. First, owing to its retrospective design, some cases with incomplete data were excluded, potentially introducing selection bias. Second, the single sampling point strategy employed in this study, which involved collecting samples 2 h after administration, may not accurately capture the true peak concentration in all individuals owing to inter-individual variability in pharmacokinetics. Third, this study focused exclusively on Asian populations, limiting the generalizability of the findings to other ethnic groups. Further studies involving diverse populations are required to validate these findings. Finally, we found a history of alcohol use increases the likelihood of neuropsychiatric AEs in patients on Cs, but we cannot quantify alcohol consumption. If accurate quantification of alcohol consumption were achieved, the study’s findings would attain greater clinical and epidemiological significance.

### Conclusions

Results from this study suggest that Cs *C*_max_ serves as a critical indicator for predicting Cs-related neuropsychiatric ADRs, with a higher *C*_max_ correlating with an increased likelihood of neuropsychiatric ADRs. Dynamic monitoring of Cs *C*_max_ combined with alcohol use history assessment in clinical practice may enable the accurate identification of high-risk populations for neuropsychiatric ADRs, thereby providing a scientific basis for developing individualized medication regimens and implementing early interventions.

## SUPPLEMENTAL MATERIAL

Table S1 spectrum.01437-25-s0001.pdf Cycloserine Neuropsychiatric Adverse Reaction Assessment Scale.

ASM does not own the copyrights to Supplemental Material that may be linked to, or accessed through, an article. The authors have granted ASM a non-exclusive, world-wide license to publish the Supplemental Material files. Please contact the corresponding author directly for reuse.
