# Supplementary material for: Cycloserine-induced neuropsychiatric toxicity in multidrug-resistant tuberculosis: association with peak plasma concentration
Source: Microbiol Spectr. 2025 Nov 5;13(12):e01437-25. doi: 10.1128/spectrum.01437-25 (PMC12671155; doi:10.1128/spectrum.01437-25)
Supplement: Table S1 — Cycloserine Neuropsychiatric Adverse Reaction Assessment Scale. [file spectrum.01437-25-s0001.pdf]

Cycloserine Neuropsychiatric Adverse Reaction Assessment Scale

(1) I feel more nervous and anxious than usual ☐

(2) I experience unexplained fear or even panic ☐

(3) I frequently feel palpitations and sometimes have trembling hands or limbs ☐

(4) I have difficulty falling asleep and experience poor sleep quality ☐

(5) I often feel depressed or in low spirits ☐

(6) I frequently feel fatigued and have a loss of appetite ☐

(7) Routine tasks have become more difficult for me ☐

(8) I have lost interest in activities I normally enjoy ☐

(9) I frequently feel like crying ☐

(10) I feel hopeless about the future and sometimes question the meaning of life ☐

Evaluation Criteria (Contraindications and Precautions):

1. **Use with caution if:**
   - ≥4 items from (1)-(7) are checked.
   - Any single item from (8)-(10) is checked.
   - Psychiatric consultation is recommended before initiation, with close monitoring during treatment.
2. **Contraindicated in:**
   - Patients currently receiving anxiolytic or antidepressant therapy.
